# Tool Wear Prediction Based on Artificial Neural Network during Aluminum Matrix Composite Milling ^†^

**DOI:** 10.3390/s20205798

**Published:** 2020-10-13

**Authors:** Martyna Wiciak-Pikuła, Agata Felusiak-Czyryca, Paweł Twardowski

**Affiliations:** Faculty of Mechanical Engineering, Institute of Mechanical Technology Poznan, University of Technology, 3 Piotrowo St., 60-965 Poznań, Poland; agata.z.felusiak@doctorate.put.poznan.pl (A.F.-C.); pawel.twardowski@put.poznan.pl (P.T.)

**Keywords:** artificial neural network, tool wear prediction, aluminum matrix composite

## Abstract

This article deals with the phenomenon of tool wear prediction in face milling of aluminum matrix composite materials (AMC), class as hard-to-cut materials. Artificial neural networks (ANN) are one of the tools used to predict tool wear or surface roughness in machining. Model development is applicable when regression models do not give satisfactory results. Because of their mechanical properties based on SiC or Al_2_O_3_ reinforcement, AMCs are applied in the automotive and aerospace industry. Due to these materials’ abrasive nature, a three-edged end mill with diamond coating was selected to carry out milling tests. In this work, multilayer perceptron (MLP) models were used to predict the tool flank wear VB_B_ and tool corner wear VB_C_ during milling of AMC with 10% SiC content. The signals of vibration acceleration and cutting forces were selected as input to the network, and the tests were carried out with three cutting speeds. Based on the analysis of the developed models, the models with the best efficiency were selected, and the quality of wear prediction was assessed. The main criterion for evaluating the quality of the developed models was the mean square error (MSE) in order to compare measured and predicted value of tool wear.

## 1. Introduction

Aluminum matrix composites (AMCs) are a class of hard-to-cut metal matrix composite materials. The ingredients of AMCs are Al alloys with the most popular reinforcement materials such as SiC, Al_2_O_3_, B_4_C, and TiC. A combination of Al alloy with diverse reinforcement gives a unique blend of mechanical properties [1,2]. The reinforcement can take the separate form particulates, particulate complexes, continuous fibers, short fibers, or whiskers. The strength of these materials depends upon grain size and microstructure. AMCs offer better stiffness values and strength, lower weight, and thermic expansion coefficients compared to monolithic alloy. For example, the SiC reinforcement improves the density, hardness, tensile strength, and wear resistance of Al alloys. According to [3,4], revealed that reducing the Al_2_O_3_ size affects the increase of Al matrix composite wear resistance. Kumar [5] described that, during studies of AMC with Al_2_O_3_ particulates, the hardness of composites and tensile strength increased, but the relative elongation decreased. Particulate-based metal matrix composites (PMMCs) prove lower anisotropy and higher ductility than MMCs with fibers. These materials are made by dispersing the reinforcements in the metal matrix. The fabrication process also affects stiff particle reinforcement distribution in the metal matrix; one of the manufacturing methods is vertical pressure casting or squeeze casting. Another manufacturing method is the Rheo Casting Process that improves the distribution of the reinforcement in the matrix. This method reduces the risk of an uneven grouping of hard particles in the matrix, therefore improving the material’s ductility. Because of their mechanical properties, non-ductile behavior, and anisotropy, PMMCs are applied in the automotive and aerospace industries, military defense and nuclear industries. The MMC materials can be applied as potential lightweight materials in aerospace components. Nowadays, these materials fulfill the requirements in engineering, such as a better ratio of strength to weight and high stiffness. There is a popular alternative to traditional solutions [6,7].

During the machining of hard-to-cut materials, technological problems are occurring. One of the most common issues is shorter tool life, excessive tool wear, or unsatisfactory surface integrity or higher energy requirement [8,9]. MMCs are considered hard for machining because of the abrasive reinforcement. Difficulties in machining these materials are associated with the lack of a uniform structure, abrasive properties, and high hardness of the reinforcement phases. Adhesion tool wear has often been detected during MMCs machining. The secondary adhesion is the most common mechanism related to the machining of aluminum alloys, Repeto [10] states that it also appears in MMCs machining. In this case, SiC reinforcement causes abrasion wear to the tool, and adhesion is unitary through the tool edge, creating the build-up layer (BUL). Manna [11] described that during machining of MMC (A413/15% SiC), the lower built-up edge (BUE) is formed at high speed and low cut depth. Moreover, the stable build-up edge could protect the tool from wear by abrasion. It has been observed that the feed rate has the most significant impact on tool wear, thus increasing its value produces the (BUE) formation. The reinforcement particles are hard and too abrasive for the cutting tools. During the machining, while the cutting tool engaged on the hard particles, stress and forces are suddenly increased. Then, the cutting tool leaves more pits and cracks on the particles. Simultaneously, the cutting tool shearing, the aluminum alloy stress, and force on the tool are rereleased. This process leads to the waviness of the cutting tool and reduces surface quality [12]. It is necessary to use tools with high toughness, strength, and hardness to resist the high cutting loads for machining these composite materials. Due to these materials’ abrasive nature, it is recommended to use polycrystalline diamond (PCD) brazed tools to obtain a proper tool. According to [13], during studies of Al–SiC (10/20%) composite, the PCD tool wear was investigated. Muthukrishnan et al. proved that percentage of particles in the matrix strongly influenced the tool wear. Two-body abrasion and three-body abrasion wear were found on the tool flank wear. Additionally, it is caused by released hard particles, entrapped between the tool and workpiece. On the other hand, cubic boron nitride (CBN), alumina, silicon nitride, and tungsten carbide (WC) tooling are economical choices for low volume production. For example, using the carbide tooling, low cutting speeds, and high feed rates are applied to maximize the tool life. Although PCD diamond tools are the most preferred for machining Al/SiC, the high cost associated with them limits their use [14,15].

Milling of ceramic-reinforced aluminum matrix composite is the most general and widely used machining process in the industry. Therefore, the machining process variables such as cutting speed, feed rate, and cut depth significantly affect the tool wear and the quality of the machined surface. Wang et al. [16] investigated the high-speed milling of Al/SiC/65p and state that milling speed is the most significant cutting parameter for surface roughness. According to [17] studies of tool performance during turning of Duralcan (A356/20% SiC) with Al_2_O_3_/TiC, TiN, the BUE, and flank tool wear was measured. El-Gallab revealed that the cutting parameters (the speed of cut, feed, and depth of cut) is essential in determining the amount of tool flank wear. On the other hand, Turgut et al. [18] observed that federate and cut depth are the most crucial parameter for milling MMCs. In these studies, the cutting force increases with feed rate and depth of cut, and surface quality decreases with increasing depth of cut and feed rate. Zhou et al. [19] proposed a FE (finite element) simulation based on cutting forces and equivalent stress models during machining of SiCp/Al composites at different cutting conditions. They state that cutting speed and depth of cut have significant effects on the cutting force. Monitoring or detecting tool wear and sudden tool failure are essential for improving manufacturing processes’ reliability. The monitor tool wear methods could be divided into two types: direct (optical, microscope, electrical resistance, etc.) and indirect (vibration, force, torque, acoustic emission, etc.) [20,21,22]. Basically, in tool condition monitoring (TCM), the tool state is determined by analyzing the signals from sensors**/**multi-sensors. Based on sensor signals, the correlation between feature parameters and the tool states effectively adapts [23]. The use of sensors for measuring cutting forces, acoustic emission, mechanical vibrations, or acoustic vibrations (noise) is expected. In machining, mainly strain gauges, piezoelectric sensors, and integrated and multi-component sensors are used, while fast Fourier transform (FFT) is most commonly used for digital signal processing [24,25]. Azmi [26] developed a tool condition monitoring technique based on measured machining force data and adaptive network-based fuzzy inference systems during end milling of the GFRP composites. The results revealed that the ANFIS models matched the nonlinear relationship of tool wear and feed force highly effective compared to that of the simple power law of regression trend. In addition to the right selection of tool material, unconventional machining improved cutting performance is common. One of the used methods is the electrical discharge machining (EDM), laser machining (LAM), electrochemical machining, ultrasonic machining (USM), and high-speed machining [27,28,29]. According to [30], Chwalczuk et al. shows the optimization of heating and cutting parameters during turning of Inconel 718 under laser-assisted machining (LAM) conditions. They proved that the dendritic structure appears in the laser affected zone of the Ni-based alloy for sequential LAM. Due to surface softening kind of microstructures cause better machinability of hard to cut Inconel 718.

In addition to the use of unconventional machining methods to improve MMC materials’ machinability, modern research is based on optimization methods and tools to generate a solution for engineering problems. The most frequently used optimization methods to improve the surface quality or minimize tool wear are the Taguchi method, response surface methods (RSM), adaptive network-based fuzzy inference systems (ANFISs), analysis of variance (ANOVA), and artificial neural networks (ANN) [31,32,33]. According to [34] studies, Basheer et al. investigated the surface roughness prediction model in precise machining of MMCs using PCD tools considering volume and size of reinforcement, tool nose radius or feed rate, and depth cut based on ANN. Arokiadass et al. [35] developed the empirical relationship to predict tool flank wear during end milling of Al/SiCp composites considering process parameters. The developed model (using ANOVA analysis) effectively predicts the tool flank wear of carbide end mill at 95%. Based on researches, the cutting parameter with the most significant impact on tool wear or surface roughness can be found. For example, Karabulut et al. [36] observed improvement of the surface roughness Ra during milling of AMCs using higher cutting speeds and lower feed rates. In this study, the experiment was performed based on the Taguchi method, and ANN evaluated the prediction error. Four layers network was constructed to predict the optimal output data in the propagation phase. The prediction model was developed with the prediction performance of over 97%. The effectiveness of the model results from the usefulness of the ANN in difficult to cut materials milling. In Chandrasekaran et al. [37] work, ANN was applied to the surface roughness prediction model during cylindrical grinding of LM25/SiC/4p MMC. 4-12-1 ANN model with logistic transfer function was created with 94.20% prediction accuracy. The independent input machining parameters on surface roughness were checked with the percentage of wheel velocity contribution −32.47%, feed −26.50%, and workpiece velocity −25.08%. Moreover, Devarasiddappa et al. [38] describe the surface roughness prediction model in end milling of Al–SiCp MMC using ANN. In this investigation, the average error of predictive performance equals 0.31% against 0.53% using RMS models. According to [39], Tsao et al. investigated the radial basis function network (RBFN) and the Taguchi’s method with three factors (spindle speed, feed rate, and drill diameter) to predict surface roughness and thrust force in the drilling of WFC200 fabric carbon fiber/epoxy matrix (CFRP). The correlations were received by RBFN and multi-variable regression analysis and compared with experimental data. In general, RBFN is more effective than the multi-variable regression analysis. In Marani et al. [40] research, various adaptive network-based fuzzy inference systems (ANFISs) were applied to predict surface roughness and cutting force during milling of Al-20Mg2Si MMC. The authors selected the two most precise models, and as a result, the root means square error (RMSE) value of surface roughness predicting was 0.2846 and 2.4053 for cutting force. It also means that these models can significantly predict the machinability of MMC. In paper [41], Wu et al. developed the convolutional neural network (CNN) model to automatically identify tool wear during the face milling process of high-temperature alloy Inconel 718. To pre-train, the network model convolutional automatic encoder (CAE) was used. In these studies, the experimental results indicate the model’s average recognition precision rate at the level of 96.20%.

The problem of MMCs milling and real-time tool wear assessment is still significant, so optimization and predictive solutions are continually being sought. In most of the work on the machinability of AMCs, the prediction models are based on cutting parameters, size of reinforcement, or cutting tool parameters. There is a lack of work focused on prediction models based on cutting forces and acceleration of vibrations signals during milling of hard to cut Al/SiC (10%) composite. The aim of research involved the diagnosis of tool wear, based on the vibration acceleration and cutting force measurement during end milling of difficult-to-cut AMC with 10% SiC content. The research’s essential element was checking the effectiveness of diagnosing the tool condition based on the developed artificial neural networks (ANN) models. For this purpose, MLP networks with different activation functions were selected based on cutting force and vibration acceleration measures in the time domain and frequency domain. Testing models of various structures allowed establishing the most effective networks for predicting tool flank and corner wear.

## 2. Materials and Methods

**Figure 1:**
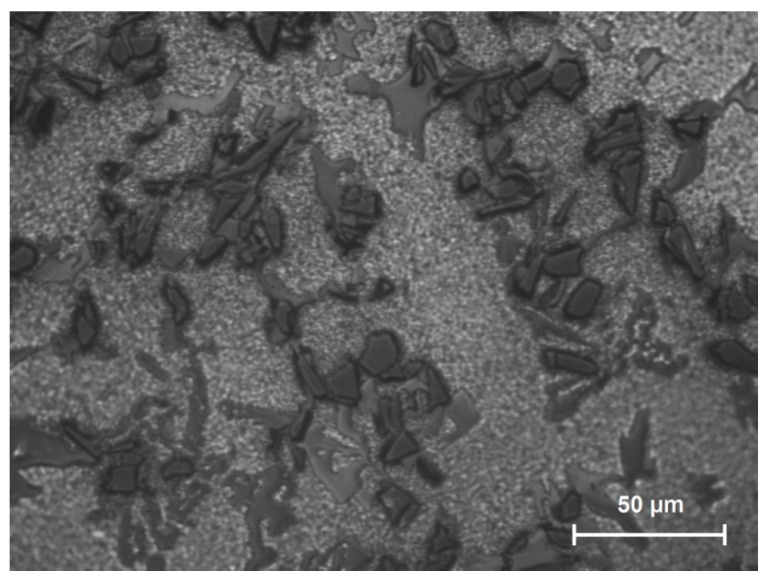
The metallographic microsections of the Al/SiC (10%).

The study used a hard-to-cut material, the Al/SiC matrix composite, as a workpiece. The reinforcement of aluminum cast alloy with the silicon carbide particles (approximately 10% SiC) improves mechanical properties. The metallographic microsections of the AMC composite is shown in Figure 1. Moreover, Table 1 depicts the chemical decomposition of the Al/SiC composite workpiece. A three-edge end mill with diamond coating was selected to carry out milling tests. Table 2 shows the tool characteristics.

The cutting tests were conducted on the DECKEL-MAHO DMC 70 V machining center. The cutting speed *v_c_* was one variable parameter in tests. To check the repeatability of the measurements, three repetitions were carried out for each cutting speed. Table 3 presents the research plan.

The tests were carried out as follows. During each milling pass, the vibration acceleration and cutting force were measured. Additionally, after each tenth pass, the tool flank wears *VB_B_*, and the tool corner wear *VB_C_* was inspected using a microscope. The tool wear criterium *VB_imax_* was equal to 0.3 mm.

During the end milling operation of Al/SiC composite, the following cutting force components were measured in three directions:

- *Ff (Y)* for feed direction;
- *FfN (X)* for normal feed direction;
- *Fp (Z)* for the axial direction.

Also, the acceleration of vibration was measured in the following different directions:

- *Af (X)* for feed direction;
- *AfN (Y)* for normal feed direction;
- *Ap (Z)* for axial direction.

Triaxial piezoelectric charge accelerometer Type 4321 Brüel and Kjær was selected to measure vibrations in three independent directions during research. This accelerometer is suited to operate temperatures up to 250 °C and measure up to 10,000 Hz. This piezoelectric accelerometer was attached to the MMC workpiece. Table 4 depicts the specifications of the 3D piezoelectric accelerometer.

**Figure 2:**
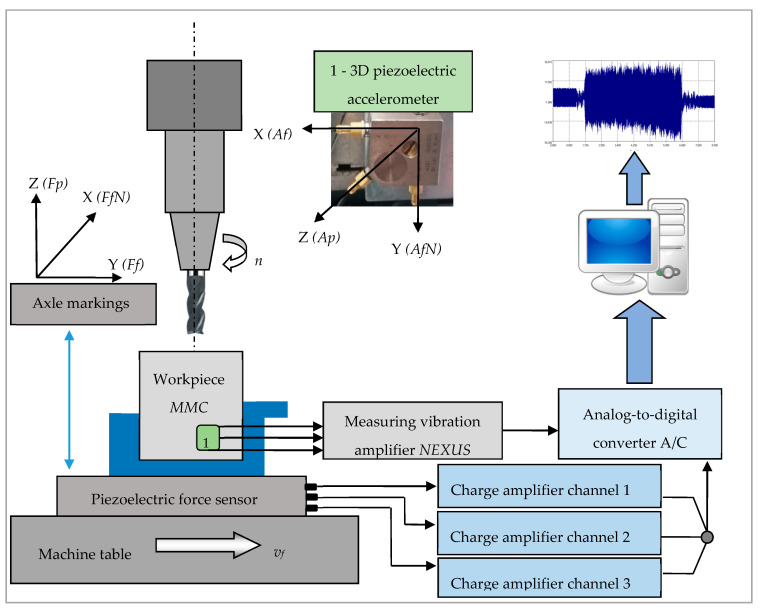
Scheme of experimental set up.

Measuring of cutting forces was carried out using a piezoelectric force sensor, and processing of signals was conducted with the use of Kistler Charge Meter Type 5015A. Table 5 shows the parameters of the piezoelectric dynamometer. In research, three charge meters have been applied. Each of them was applied in a different direction: X, Y, and Z. Figure 2 shows the scheme of the experimental apparatus set up.

## 3. Results

### 3.1. Analysis of Tool Wear 

The first step in analyzing results is to analyze measured tool flank wear *VB_B_* and tool corner wear *VB_C_*. In order to determine the relations between the tool wear and cutting time, a third degree polynomial function was selected as the most representative for the tool wear process:

(1) $$VB_{i}=a\cdot t_{c}^{3}+b\cdot t_{c}^{2}+c\cdot t_{c}$$

where *VB_i_*—tool wear, *t_c_*—cutting time.

**Figure 3:**
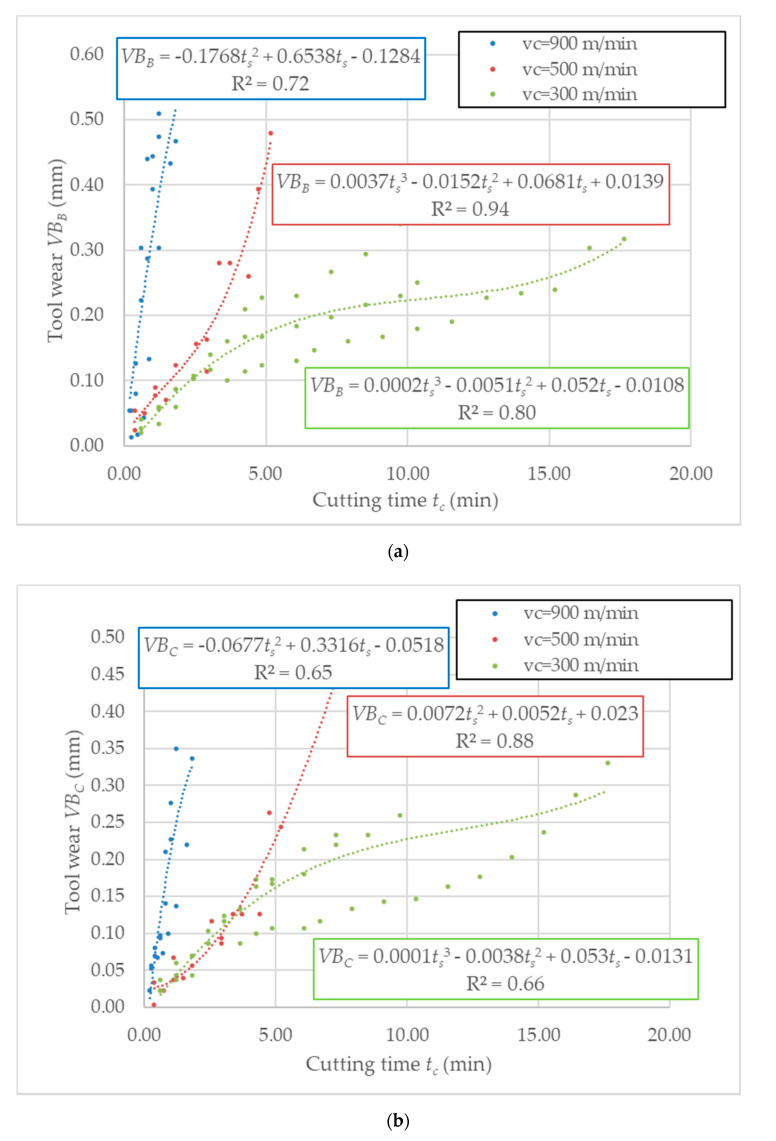
Tool wear, as a function of cutting time *t_s_*: (**a**) tool corner wear *VB_C_*, (**b**) flank wear *VB_B_*.

Analysis for the corner wear *VB_C_* and the flank wear *VB_B_* was carried out separately. The tool wear criterion in both cases was 0.3 mm. The coefficient R^2^ indicates an adjustment to the selected mathematical function. Figure 3 depicts tool flank wear *VB_B_* and *VB_C_* values in function of time for all repetitions.

Based on the determined equation, the average time needed for excessing the critical tool wear value has been calculated and shown in Table 6. Above this value, the unequal tool wear is caused by tool wear adhesion detected during AMC composite machining.

The tool wear is significant in all cases; the loss of tool material and the material adhered in the corner is visible, mainly after milling with *v_c_* = 900 m/min. While the cutting tool engaged in the hard particles, stress, and forces are suddenly increased. Then, cutting edge chipping is occurs. During machining of AMC material, the secondary adhesion appears in an investigation, as it is a thermomechanical mechanism. However, due to stiff SiC reinforcement, the abrasion wear occurs and provokes flank wear. Almost throughout the tool edge, adhesion is visible, creating an adhered layer or BUL (built-up layer). Additionally, the increases of abrasion and flank wear are caused by these hard particles. Furthermore, due to friction, the rise in temperature is expected, which results in very short cutting time. In Table 7, the tool corner wear of the tool in various cutting conditions is shown. Moreover, the state of flank wear is presented in Table 8.

### 3.2. Analysis of Vibration in Time and Frequency Domain

**Figure 4:**
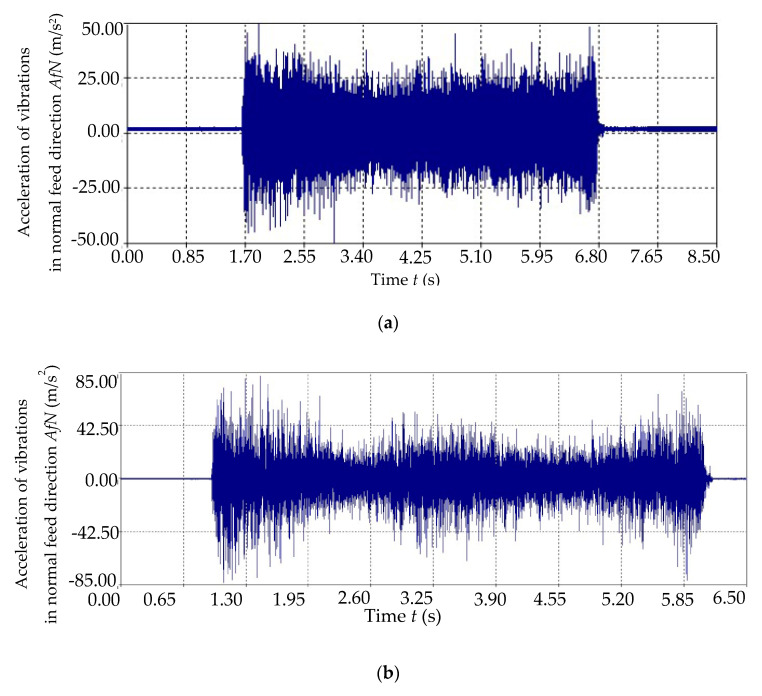

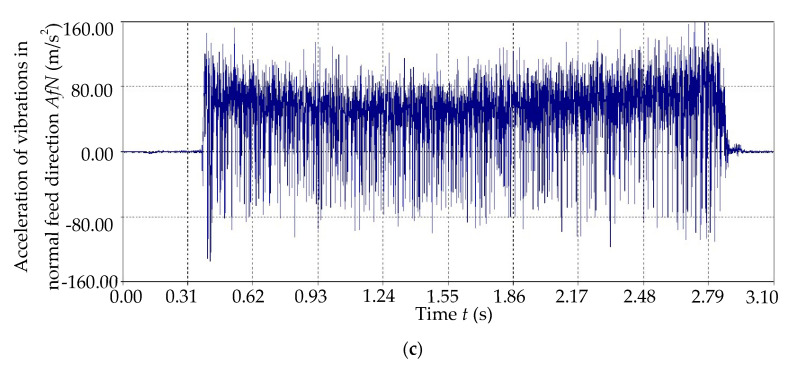
Vibration time course for (**a**) *v_c_* = 300 m/min, (**b**) *v_c_* = 500 m/min, (**c**) *v_c_* = 900 m/min.

**Figure 5:**
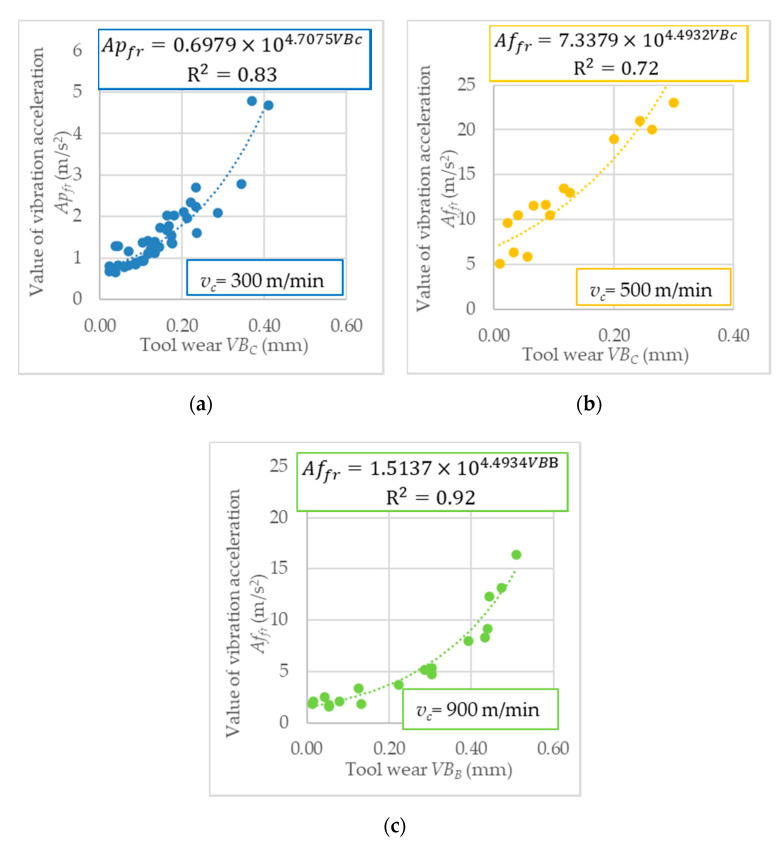
Diagnostic measures of vibration acceleration’s in: (**a**) frequency domain, *v_c_* = 300 m/min; (**b**) time domain, *v_c_* = 500 m/min; and (**c**) frequency domain, *v_c_* = 900 m/min; as a function of tool wear.

The next step after the conducted tests was to correlate the tool wear values with measured accelerations of vibrations in the frequency domain and time domain. Based on the generated charts in “Analyzer” software, the tooth passing and tool revolution frequencies were identified. For cutting speeds applied in investigation, the tool revolutions frequencies are: *Fr*_300_ = 477 Hz, *Fr*_500_ = 796 Hz and, *Fr*_900_ = 1433 Hz. Figure 4 depicts the exemplary vibration chart in the time domain. Moreover, in Figure 5, the relation between the diagnosis measures of accelerations of vibrations and tool wear based on milling repetition in various cutting speed are presented.

Based on vibration acceleration, the best results were obtained for the exponential function for all diagnostic measures. The R^2^ coefficient represents the matching of the assumed mathematical function to the experimental results. Table 9 shows R^2^ values of diagnosis measures analyzed in the frequency domain (*Ai_fr_*), and root means square value (RMS) based on the time domain.

### 3.3. Analysis of Cutting Forces

**Figure 6:**
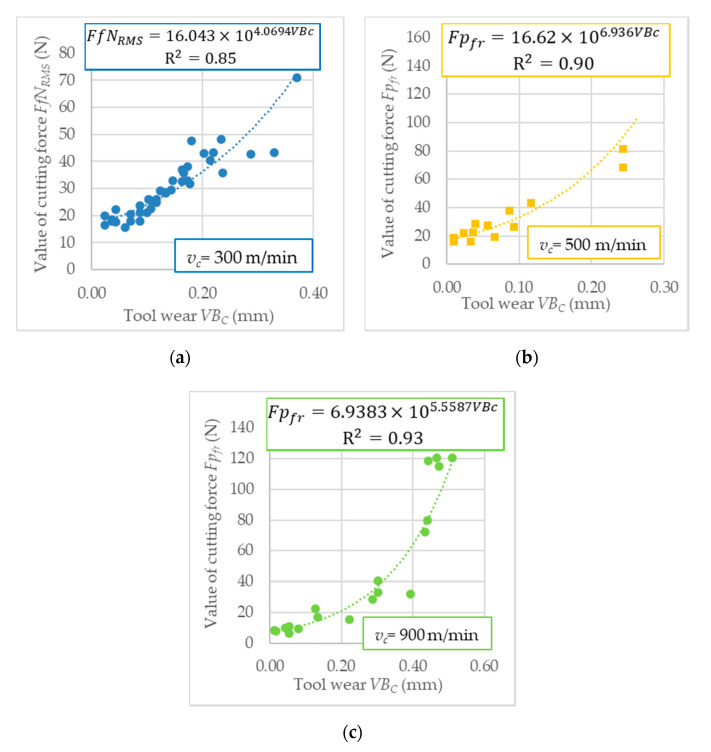
Diagnostic measures of cutting force components in (**a**) time domain, *v_c_* = 300 m/min; (**b**) frequency domain, *v_c_* = 500 m/min; and (**c**) frequency domain, *v_c_* = 900 m/min; as a function of tool wear.

The next step was to recognize the relationship between the tool wear and cutting force components measure as a diagnostic signal. Figure 6 depicts the correlation between diagnostic measures and tool wear. The R^2^ coefficient represents the matching of the exponential mathematical function to the experimental results. Table 10 depicts R^2^ values of diagnostic measures analyzed in the frequency domain (*Fi_fr_*), and root means square value (RMS) based on the time domain.

### 3.4. Diagnostic Model Based on Artificial Neural Network

**Figure 7:**
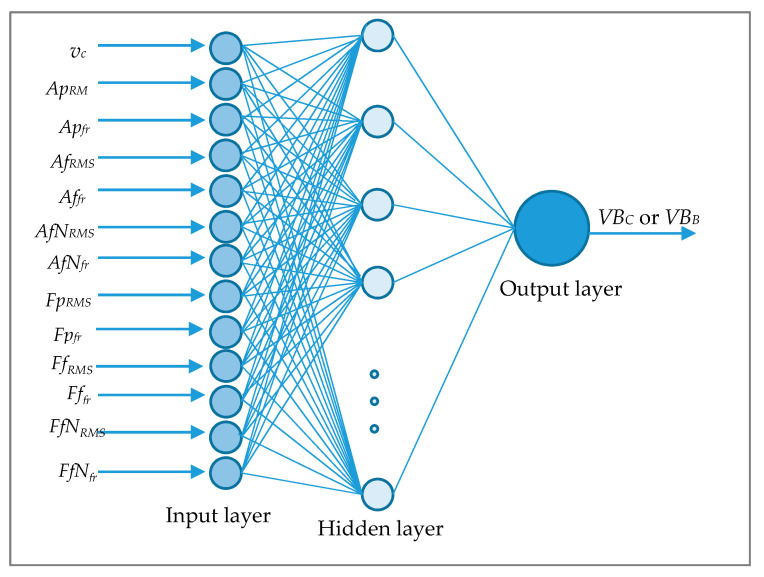
Structure of multilayer perceptron (MLP) network for tool wear prediction.

Using ANN in this work was to assess the possibility of predicting tool conditions based on measured signals. The low correlation coefficient between tool wear and diagnostic measures indicates the chances of using other methods than regression models. During this work, a multilayer perceptron (MLP) was used to check the tool wear prediction effectiveness. The input data were diagnostic measures based on the analysis of the cutting forces components and vibration acceleration. One of the steps was focused on learning the neural networks using the Broyden–Fletcher–Goldfarb–Shanno algorithm (BFGS), which is considered one of the most effective. The (BFGS) training algorithm, i.e., back propagation, is characterized by high stability and low error sensitivity [21]. Due to select the best ANN, the activation functions were changed in the hidden and the initial layer: linear, logistic, tangent, and exponential. Figure 7 shows the scheme of structure two-layered MLP network model with thirteen of inputs.

Searching for the model with the most effective quality of ANN network testing for *VB_C_* and *VB_B_* prediction, the MLP networks were selected with different activation functions and with the least validation errors. The 50 models were checked, and fourteen models with the best testing efficiency were selected. Table 11 presents the characteristics of selected MLP networks for corner tool wear prediction. The number of random samples was assumed at 70% for the training set, 15% for the test set, and 15% for the validation set. The number of epochs for data was 200. The network name means, for example, MLP 13-1-1: 13—input data, 1—hidden layer, and 1—output.

**Figure 8:**
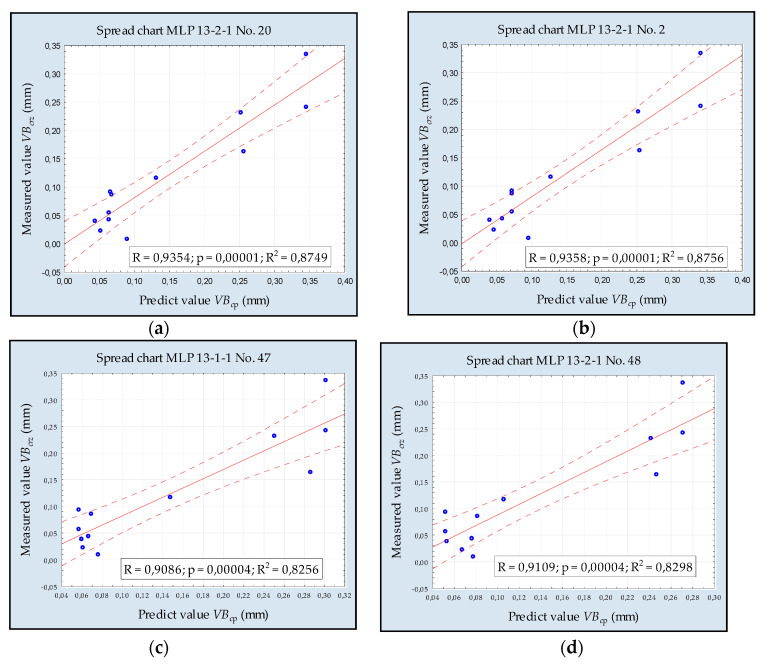
Verification model of (**a**) MLP No. 20, (**b**) MLP No. 2, (**c**) MLP No. 47 and, (**d**) MLP No. 48.

Based on new experimental data, the mean square error (MSE) was estimated to compare measured and the predicted value of *VB_C_*. Figure 8 shows a spread graph of these values for neural network MLP with the best validation quality for four models with the lower MSE.

**Figure 9:**
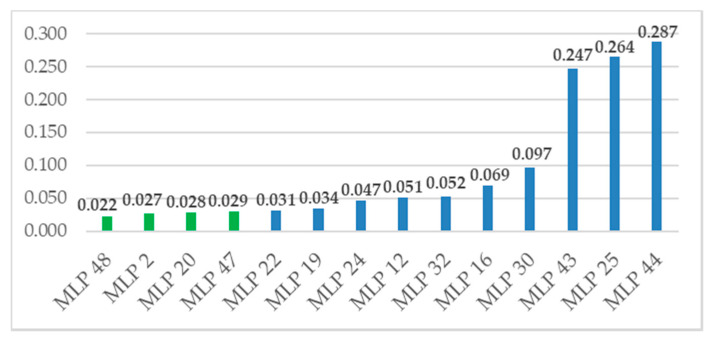
Value of mean square error (MSE) error for verification artificial neural networks (ANN) models.

The main criterion for assessing the developed models’ quality was to compare the MSE error for new data. These experimental data were not entered into the network learning process. Figure 9 presents a comparison of the errors of individual MLP networks. The highest error values were observed when the number of neurons in the hidden layer was greater than or equal to nine.

## 4. Conclusions

Based on the experimental results of the present work, the following conclusions can be drawn.

- Analysis of the correlation of the diagnostic measures used, such as vibration acceleration or cutting force with tool wear, shows an unsatisfactory correlation coefficient R^2^. During normal wear of the cutting tool, higher vibration signals appear over time, which can be caused by encountering hard SiC particles in the material’s matrix.
- The use of artificial neural network models with thirteen inputs has significantly improved the correlation coefficient’s value. The effectiveness of predicting wear based on forces and vibrations gave satisfactory results, and the lowest MSE error between the measured and predicted data was 0.022.
- Based on the models, it can be seen that the tangent and logistic activation function in the hidden layer gave the best results in the learning and testing process. However, one or two neurons in the hidden layer bring the significant effects of tool wear prediction. Increasing the number of neurons in the hidden layer does not improve the performance of prediction models.

To sum up, the use of ANN models to predict tool wear during AMC materials milling is a significant prediction tool. Developed MLP models based on specific cutting conditions with input parameters: cutting speed, cutting forces, and accelerations of vibrations are satisfactory to predict tool wear. Based on these signals, effective prediction of tools with diamond coating wear’s during end milling of hard-to-cut Al–SiC (10%) composite is allowed (average value of MSE equals 0.027); This enables identifying tool wear’s criterium during machining.

## Figures and Tables

**Table 1:** The chemical composition Al/SiC composite.

Element	Si	Fe	Cu	Mg	Ti	Al
[%]	8.5–9.5	0.2 max	0.2 max	0.45–0.65	0.2 max	rest

**Table 2:** Characteristic of three-edge end mill.

Diameter of the Cutting Edge *d*_1_ (e8) (mm)	Shank Diameter *d*_2_ (h6) (mm)	Total Length of the Tool *l*_1_ (mm)	Length of Cutting Edge *l*_2_ (mm)	Corner Radius *r* 0/+0.03 (mm)
10	10	72	22	0.5

**Table 3:** Research plan with one variable.

Cutting Speed *v_c_* (m/min)	Feed per Tooth *f_z_* (mm/tooth)	Feed Rate *v_f_* (mm/min)	Spindle Speed *n* (rev/min)	Axial Infeed Depth *a_p_* (mm)	Radial Infeed Depth *a_e_* (mm)
300	0.035	1003	9544	8	0.2
500		1671	15,923		
900		3009	28,662		

**Table 4:** Specifications of piezoelectric charge accelerometer.

Sensitivity (pC/ms^−2^)	Operating Temperature Range (°C)	Capacitance (pF)	Linear Frequency Range (Hz)
1.0	−74 to +250	1100	0.1–10000

**Table 5:** The measurements parameters of piezoelectric dynamometer.

	Measuring Range (N)	Frequency (kHz)	Sensor Sensitivity (pC/N)
X,Y	1000	30	−8.795
Z	1000	30	−2.370

**Table 6:** Average value of tool life for various cutting speed.

	900 (m/min)	500 (m/min)	300 (m/min)
*t_s_critical_* (min)	≈2.2	≈5	≈16

**Table 7:** State of tool corner wear *VB_C_* after milling with various cutting speed.

	Tool Edge I	Tool Edge II	Tool Edge III
300 m/min	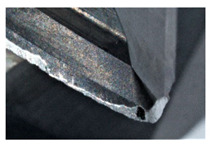	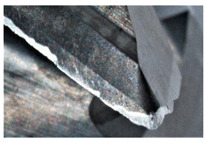	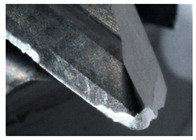
500 m/min	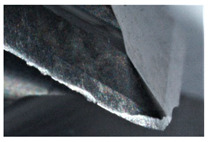	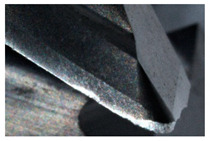	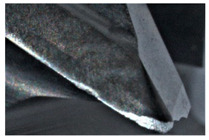
900 m/min	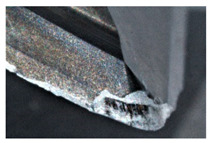	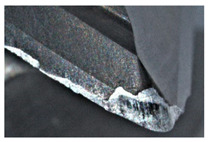	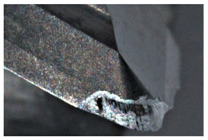

**Table 8:** State of flank wear *VB_B_* after milling with various cutting speed.

	Tool Edge I	Tool Edge II	Tool Edge III
300 m/min	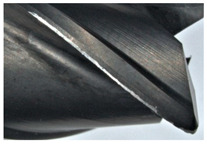	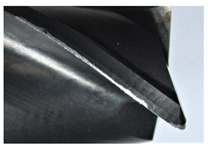	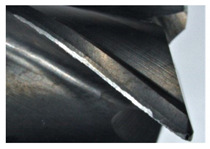
500 m/min	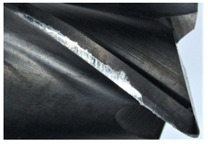	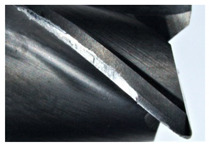	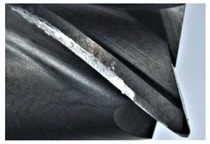
900 m/min	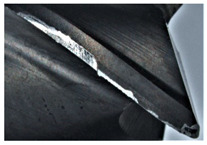	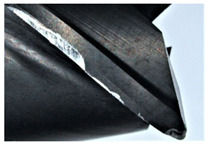	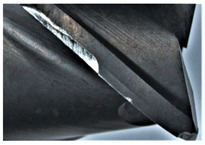

**Table 9:** Values of coefficient R^2^ based on vibration acceleration.

Diagnostic Measure	300 m/min	500 m/min	900 m/min
*Af_fr_*	0.77	0.72	0.92
*Ap_fr_*	0.83	0.82	0.87
*AfN_fr_*	0.83	0.78	0.83
*Af_RMS_*	0.61	0.81	0.70
*Ap_RMS_*	0.61	0.74	0.75
*AfN_RMS_*	0.70	0.92	0.84

**Table 10:** Values of coefficient R^2^ based on cutting forces.

Diagnostic Measure	300 m/min	500 m/min	900 m/min
*Ff_fr_*	0.78	0.86	0.63
*Fp_fr_*	0.71	0.90	0.93
*FfNc_fr_*	0.81	0.84	0.84
*Ff_RMS_*	0.79	0.74	0.7
*Fp_RMS_*	0.81	0.75	0.61
*FfN_RMS_*	0.85	0.88	0.78

**Table 11:** Structure of MLP networks (*VB_c_*).

ANN Name	Educational Quality	Testing Quality	Validation Quality	Activation Function (HL)	Activation Function (OUT)
1.MLP 13-2-1	0.943812	0.913454	0.666538	Logistic	Logistic
2.MLP 13-2-1	0.943384	0.965668	0.923702	Logistic	Tangent
3.MLP 13-3-1	0.940617	0.904863	0.798475	Exponential	Logistic
4.MLP 13-5-1	0.984513	0.958691	0.791474	Exponential	Logistic
5.MLP 13-5-1	0.943914	0.959692	0.445910	Logistic	Exponential
6.MLP 13-4-1	0.842717	0.971133	0.818754	Exponential	Logistic
7.MLP 13-5-1	0.979490	0.952491	0.820367	Exponential	Tangent
8.MLP 13-5-1	0.959143	0.841588	0.733929	Tangent	Tangent
9.MLP 13-3-1	0.940015	0.922483	0.583598	Logistic	Tangent
10.MLP 13-5-1	0.952375	0.939408	0.807712	Tangent	Logistic
11.MLP 13-4-1	0.970583	0.952569	0.479804	Tangent	Tangent
12.MLP 13-3-1	0.963558	0.937907	0.899602	Logistic	Logistic
13.MLP 13-2-1	0.894731	0.968126	0.566312	Linear	Linear
14.MLP 13-5-1	0.982973	0.990609	0.718583	Logistic	Linear
15.MLP 13-2-1	0.923793	0.958984	0.861170	Tangent	Exponential
16.MLP 13-1-1	0.912820	0.908474	0.941118	Linear	Logistic
17.MLP 13-5-1	0.946393	0.961313	0.584911	Tangent	Exponential
18.MLP 13-3-1	0.966975	0.958082	0.717795	Tangent	Linear
19.MLP 13-3-1	0.967312	0.950368	0.931474	Tangent	Tangent
20.MLP 13-2-1	0.935502	0.954024	0.860694	Logistic	Tangent
21.MLP 13-7-1	0.898268	0.920376	0.879598	Logistic	Logistic
22.MLP 13-7-1	0.965828	0.943079	0.970559	Logistic	Tangent
23.MLP 13-8-1	0.954264	0.934399	0.883744	Logistic	Logistic
24.MLP 13-9-1	0.933910	0.930772	0.913040	Logistic	Tangent
25.MLP 13-9-1	0.972380	0.959144	0.920862	Exponential	Exponential
26.MLP 13-6-1	0.971846	0.988926	0.763942	Logistic	Linear
27.MLP 13-10-1	0.965716	0.944992	0.899398	Exponential	Linear
28.MLP 13-6-1	0.923811	0.783621	0.683669	Linear	Exponential
29.MLP 13-10-1	0.948735	0.939010	0.832663	Tangent	Tangent
30.MLP 13-10-1	0.919947	0.945050	0.903948	Exponential	Exponential
31.MLP 13-7-1	0.894533	0.969097	0.573019	Linear	Linear
32.MLP 13-6-1	0.944073	0.939564	0.932381	Logistic	Logistic
33.MLP 13-10-1	0.915916	0.780465	0.733684	Linear	Exponential
34.MLP 13-6-1	0.956536	0.974358	0.863555	Exponential	Linear
35.MLP 13-6-1	0.945611	0.903929	0.719954	Linear	Logistic
36.MLP 13-6-1	0.952249	0.935221	0.877035	Exponential	Linear
37.MLP 13-9-1	0.959055	0.917057	0.861010	Logistic	Logistic
38.MLP 13-9-1	0.951087	0.936644	0.884964	Logistic	Tangent
39.MLP 13-8-1	0.950654	0.897484	0.802463	Tangent	Tangent
40.MLP 13-6-1	0.871946	0.924307	0.734561	Exponential	Logistic
41.MLP 13-11-1	0.941935	0.953774	0.826152	Tangent	Linear
42.MLP 13-11-1	0.971210	0.908395	0.696591	Tangent	Linear
43.MLP 13-11-1	0.947782	0.972040	0.878610	Tangent	Linear
44.MLP 13-11-1	0.959047	0.968386	0.944422	Tangent	Linear
45.MLP 13-11-1	0.940225	0.967683	0.852926	Tangent	Linear
46.MLP 13-11-1	0.977849	0.959445	0.696598	Tangent	Linear
47.MLP 13-1-1	0.929770	0.919586	0.944269	Tangent	Exponential
48.MLP 13-2-1	0.954401	0.977467	0.605264	Tangent	Exponential
49.MLP 13-13-1	0.958803	0.968876	0.840884	Tangent	Linear
50.MLP 13-13-1	0.957613	0.953265	0.940080	Tangent	Linear
